# Homocysteine Is a Risk Factor in Predialysis Patients When Associated with Malnutrition and Inflammation

**DOI:** 10.4061/2010/957645

**Published:** 2010-06-21

**Authors:** Ana de Lurdes Agostinho Cabrita Vieira, Alexandre Baptista, Anabela Malho, Ana Pinho, Ana Paula Silva, Idalécio Bernardo, Pedro Leão Neves

**Affiliations:** Serviço de Nefrologia, Hospital de Faro, Algarve, Rua Leão Penedo, 8000-342 Faro, Portugal

## Abstract

The increased level of plasma total homocysteine (tHcy) in chronic kidney disease patients has been reported as a new and independent risk factor for cardiovascular disease. However, after the description of reverse epidemiology in the renal population, the association of tHcy and nutrition became less clear. We evaluated the association between homocysteine, nutritional status, and inflammation, and their impact on mortality in 95 predialysis patients. High sensitivity C-Reactive Protein (hs-CRP), interleukin 6 (IL-6), Tumor Necrosis Factor *α* (TNF-*α*)], and tHcy were evaluated, as was the nutritional status by the modified Subjective Global Nutritional Assessment (mSGA). We divided our population in four groups according to their tHcy and mSGA values being above or below the mean level and found the lowest survival in the group with tHcy and mSGA above the mean level, as well as higher levels of IL-6 (*P* = .03) and TNF-*α* (*P* = .045). Higher levels of homocysteine can be associated with higher mortality in predialysis patients, as long as they are associated with malnutrition and inflammation.

## 1. Introduction

Homocysteine (Hcy) is an intermediate of methionine metabolism. Hyperhomocysteinemia is frequent among patients with chronic renal insufficiency, although the underlying cause is not clearly understood. It seems to involve reduced clearance of plasma Hcy, witch may be attributable to defective renal clearance and/or extrarenal clearance and metabolism, the latter possibly due to retained uremic inhibitory substances [1].

Increased levels of Hcy have been associated to increased overall mortality and to high mortality from cardiovascular disease (CVD) [2, 3]. All except one study [4] described some relation between vascular disease and Hcy levels. There is also evidence that Hcy can alter the coagulation system and the resistance of the endothelium to thrombosis [5]. It also interferes with the antithrombotic and coagulation functions of nitric oxide [6].

Recent studies have reported a reversed epidemiology in chronic kidney disease (CKD) patients where low, rather then high plasma Hcy is an indicator of poor outcome [7–10]. However, this association remains unclear because wasting and inflammation seem to share the responsibility for this reverse association, as they both decrease serum albumin levels, witch are a major determinant of Hcy levels [8]. In addition they are risk factors for increased morbidity and mortality [8–11]. The aim of the present study was to evaluate the association between Hcy levels, nutritional status and inflammation, and their impact on mortality in predialysis kidney patients.

## 2. Patients and Methods

We prospectively followed 95 patients at our low-clearance outpatient clinic at Nephrology Department of Hospital de Faro from August 2003 to August 2006. Informed consent was obtained from all patients. At baseline, a complete clinical history and a physical examination were performed. Patients were considered to have hypertension if sitting blood pressure (BP) was at least 140/90 mmHg or, regardless of BP, if they were receiving antihypertensive therapy.

Fasting blood samples were collected to measure hemoglobin, albumin, creatinine, triglycerides, and cholesterol (total and HDL).

The glomerular filtration rate (GFR) was calculated according to the Modification of Diet in Renal Disease (MDRD) equation.

Plasma, collected using heparin as the anticoagulant, was separated within 30 minutes of drawing and stored at −80°C until analysis for hs-CRP, IL-6, TNF-*α*, and tHcy (chemiluminescent assay) were performed. The modified Subjective Global Nutritional Assessment (mSGA) was used to evaluate the nutritional status.

The population was divided in 4 groups, depending if their tHcy and mSGNA values were above or below the mean level. The mean level of tHcy in our population was 25.7 *μ*mol/L and the mean value of mSGA was 12.2. The groups were as follows: Group I-tHcy <25.7 *μ*mol/L, mSGA <12.2, *n* = 29; Group II-tHcy <25.7 *μ*mol/L, mSGNA >12.2, *n* = 18; Group III-tHcy >25.7 *μ*mol/L, mSGA <12.2, *n* = 28; Group IV tHcy >25.7 *μ*mol/L, mSGNA >12.2, *n* = 20.

*Statistical analysis*—we first did descriptive statistics; values were expressed as mean ± standard deviation; to compare different groups we used the chi-square and ANOVA tests; we used the linear regression model to evaluate the relationship between variables; we used the Pearson correlation to evaluate the correlation between Hcy and inflammatory and nutritional parameters; for survival analysis we used the Kaplan-Meier method and for comparison of different groups survival we used the logrank test. The statistical analysis was performed using SPSS 11.0 for Windows (SPSS, Chicago, IL).The null hypothesis was rejected below the 5% level (*P *< .05).

## 3. Results

We included 95 patients (f = 41, m = 54), with an average age of 69.4 years, followed in our outpatient clinic for 24.1 months. The original disease in 23 patients (24.2%) was unknown; 30 patients (31.5%) had diabetic nephropathy; 19 patients (20%) had hypertensive renal disease; 15 patients (15.8%) had chronic interstitial disease, 5 (5.3%) had glomerulonephritis and 3 (3.2%) had polycystic kidney disease. The prevalence of hypertension in our study was 80% (76 patients), with 55.8% (53 patients) receiving angiotensin-converting enzyme inhibitors and/or angiotensin receptor blockers.

The characteristics of the population are showed in Table 1.

Nutritional data was obtained from modified subjective global nutritional assessment (mSGNA). Seven patients had normal nutritional status, 68 were mild malnourished and 19 were moderate malnourished, while any patient had severe malnutrition. The mean value of mSGA was 12.2 ± 3.6.

Using the Pearson correlation, we found an inverse correlation between plasma tHcy and the eGFR and a positive correlation between tHcy and hs-CRP and IL-6 (Table 2).

The four groups, divided according the tHCy and mSGA values, were compared, and the results are displayed in Table 3.

There were no significant differences between groups regarding sex distribution, and hemoglobin and CRP levels. However, Group IV (patients with higher tHcy and mSGA values) showed lower eGFR (*P* = .014), as well as higher levels of IL-6 (*P* = .03) and TNF-*α* (*P* = .045). 

During the follow-up 32 patients died: cardiovascular disease [12]; cerebrovascular disease [4]; infections [9]; neoplasia [2]; cachexia [2]; unknown [3]. 

**Figure 1:**
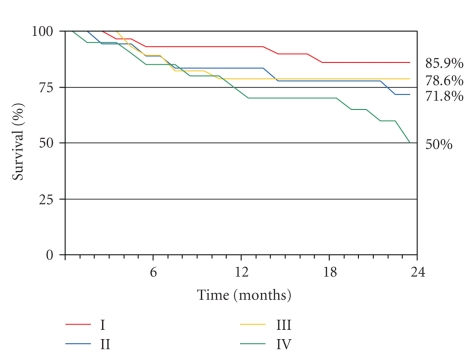
Actuarial survival at 24 months. Group IV showed the worst survival (logrank 8.31, *P* = .04).

Using the Kaplan-Meier survival analysis we found the following actuarial survival at 24 months of the different groups, as we can see in Figure 1 : I = 85.9%; II = 71.8%; III = 78.6%; IV = 50%, logrank = 8.31, *P* = .04. 

## 4. Discussion

Protein-energy malnutrition is very common in patients with chronic renal failure (CRF), with signs of malnutrition observed in 25%–40% of predialysis patients [10, 12]. We also know that the risk of death from all causes and from cardiovascular (CV) disease is increased since stage 3 of CKD [13, 14]. Classical risk factors, such as hypertension, dislipidemia or hyperuricemia, as well new risk factors, such inflammation, hyperhomocystenemia or endothelial dysfunction, contribute to this increased risk of cardiovascular mortality [15]. Hyperhomocysteinemia has attracted growing interest in recent years [10]. Epidemiological studies have shown that there is strong evidence that moderate elevation of total homocysteine levels is an independent risk factor for atherosclerosis in the general population [9, 10, 16]. Moderate hyperhomocysteinemia is present in the early stages of renal failure and increases along with the deterioration of renal function [9, 10, 17]. The prevalence of hyperhomocysteinemia is 85%–100% among patients with advanced CRF [10]. The mechanisms by which plasma Hcy levels increase in CRF are not fully understood. Reduced renal excretion and decreased renal uptake probably do not play a major role, since renal elimination of Hcy is of any significance in normal humans [17, 18]. 

Suliman et al. showed recently that high levels of homocysteine were not an independent risk factor for atherosclerosis [8]. They found that a low Hcy concentration was associated with higher all-cause and cardiovascular mortality, although this correlation could be attributed, at least in part, to the influence of various confounders, including markers of wasting and inflammation on Hcy levels [8–11]. In their work, after adjustment for all confounding factors which potentially could influence Hcy concentration, a high Hcy level showed only a trend towards a positive association with increased all-cause and cardiovascular mortality. Although not statistically significant, the all-cause and CV mortality were, respectively, 27% and 22% higher for high tHcy compared with low tHcy levels [8]. 

More recently, Kalantar-Zadeh et al., also reported that the mortality rate was higher in the lowest Hcy quartile [7]. Nevertheless, other authors have found in CKD patients that, similar to the general population, tHcy is associated with an increased risk of CV events and mortality [19].

In fact, association between low homocysteinemia and atherosclerosis does not have any theoretical plausibility, and so other factors must come into place to explain why some cardiovascular risk factors are strongly correlated with a decreased rate of cardiovascular events and death. This point of view was explored by Ducloux et al. in dialysis patients [20]. They found a positive correlation between homocysteine levels and CV mortality in dialysis patients without a chronic state of inflammation-malnutrition, and an inverse relationship between Hcy and all-cause and CV mortality in dialysis patients with chronic inflammation-malnutrition syndrome.

In our work we tried to evaluate if such association is already present in predialysis patients, and if there was any difference in mortality between the patients. We divided our population according to plasma Hcy levels and nutritional state and found that higher levels of Hcy were associated with a higher mortality rate in those patients who had a state of inflammation-malnutrition. 

These results are in contradiction with those found by Ducloux et al. probably because they used the values of CRP and albumin to define the presence of chronic inflammation-malnutrition state (CIMS). Albumin is a major determinant of tHcy levels [8], being hypoalbuminemia associated with low tHcy levels. Serum albumin levels are influenced by nutritional and nonnutritional factors [12, 21] and probably albumin is not the most accurate marker of malnutrition. To evaluate the nutritional status, we used the mSGA and in the group of patients with higher mSGA (worse nutritional status), the albumin level was higher than the value found by Ducloux et al. in their group of patients with CIMS. This fact could have determined higher tHcy levels in our patients. We also found a positive correlation between tHcy levels and markers of inflammation, namely, IL-6 and TNF-*α*. This is again in contrast with Ducloux et al. These authors found an inverse association between CRP and tHcy. Using different malnutrition and inflammation markers than Ducloux et al., we found that inflammation and malnutrition was associated with higher levels of tHcy. Furthermore, mortality was higher in patients with inflammation-malnutrition and higher tHcy. 

We believe that both factors act in synergy and that Hcy impact on mortality could not be evaluated without considering other factors, namely, inflammation. Moreover, our study shows that the impact of Hcy is dependent on the nutritional and inflammatory status.

One of the drawbacks of our study was the lack of evaluation of folate and vitamin B12 concentrations, since inadequate concentrations are contributing factors for hyperhomocysteinemia [16, 22]. More studies, with more patients, longer follow up, and wider biochemical evaluation are needed to try to answer these questions.

In conclusion, we found that higher levels of homocysteine can be associated with higher mortality in predialysis patients, as long as they are associated with malnutrition and inflammation.

## Figures and Tables

**Table 1:** Clinical, biochemical, and nutritional data.

Sex male/female	54/41
Age (years)	69.4 ± 14.4
Hemoglobin (mg/dl)	11.6 ± 1.6
Darbepoetin (*μ*g/kg/week)	0.467 ± 0.47
eGFR (ml/min/1.73 m^2^)	16.1 ± 7.0
Homocysteine, (*μ*mol/L)	25.7 ± 11.8
IL-6 (pg/mL)	5.4 ± 5.3
hs-CRP (mg/dl)	1.3 ± 2.6
TNF-*α* (pg/mL)	11.6 ± 8.3

**Table 2:** Correlation between Hcy and inflammatory/nutritional parameters and level of renal function (Pearson correlation).

	r	*P*-value
IL-6 (pg/mL)	0.289	.005
hs-CRP (mg/dl)	0.208	.043
mSGA	0.102	.323
eGFR (mL/min/1.73 m^2^)	−0.294	.004

**Table 3:** Comparison of the different groups regarding clinical and biochemical parameters.

	Group I	Group II	Group III	Group IV	*P-*value
	tHcy <25.7 *μ*mol/L,	tHcy <25.7 *μ*mol/L,	tHcy >25.7 *μ*mol/L,	tHcy >25.7 *μ*mol/L,	
	mSGA <12.2	mSGNA >12.2	mSGNA <12.2	mSGNA >12.2	
Female/male	13/16	10/8	11/17	7/13	.599
Haemoglobin (g/dl)	11.8 ± 1.5	11.7 ± 1.4	11.4 ± 1.7	11.6 ± 1.9	.827
eGFR (ml/min/1.73 m^2^)	18.8 ± 7.1	16.4 ± 6.8	16.0 ± 7.1	12.2 ± 5.4	.014
Albumin (g/dL)	4.3 ± 0.4	4.0 ± 0.5	4.3 ± 0.5	4.1 ± 0.4	.032
Hcy (*μ*mol/L)	16.5 ± 3.3	14,9 ± 4.4	32.3 ± 7.1	39.4 ± 9.0	.0001
mSGA	10 ± 1.8	15.4 ± 3.3	9.8 ± 1.6	15.9 ± 2.7	.0001
IL-6 (pg/ml)	3.3 ± 1.8	5.3 ± 3.4	5.3 ± 3.6	8.9 ± 9.3	.03
hs-CRP (mg/dl)	0.45 ± 0.6	1.3 ± 2.2	1.5 ± 2.8	2.2 ± 3.8	.122
TNF-*α* (pg/ml)	8.5 ± 3.3	11.6 ± 6.1	12.3 ± 6.3	15.1 ± 14.3	.045
